# Exploiting telerobotics for sensorimotor rehabilitation: a locomotor embodiment

**DOI:** 10.1186/s12984-021-00856-w

**Published:** 2021-04-21

**Authors:** Min Hyong Koh, Sheng-Che Yen, Lester Y. Leung, Sarah Gans, Keri Sullivan, Yasaman Adibnia, Misha Pavel, Christopher J. Hasson

**Affiliations:** 1 Department of Physical Therapy, Movement and Rehabilitation Sciences, Northeastern University, 360 Huntington Avenue, 301 Robinson Hall, Boston, MA 02115-5005 USA; 2 Division of Stroke and Cerebrovascular Diseases, Department of Neurology, Tufts Medical Center, Boston, USA; 3 Khoury College of Computer Sciences, Northeastern University, Boston, USA; 4 Departments of Bioengineering and Biology, Northeastern University, Boston, USA

**Keywords:** Teleoperation, Rehabilitation, Stroke, Locomotion, Gait, Robotics, Sensorimotor control, Motor learning

## Abstract

**Background:**

Manual treadmill training is used for rehabilitating locomotor impairments but can be physically demanding for trainers. This has been addressed by enlisting robots, but in doing so, the ability of trainers to use their experience and judgment to modulate locomotor assistance on the fly has been lost. This paper explores the feasibility of a telerobotics approach for locomotor training that allows patients to receive remote physical assistance from trainers.

**Methods:**

In the approach, a trainer holds a small robotic manipulandum that shadows the motion of a large robotic arm magnetically attached to a locomoting patient's leg. When the trainer deflects the manipulandum, the robotic arm applies a proportional force to the patient. An initial evaluation of the telerobotic system’s transparency (ability to follow the leg during unassisted locomotion) was performed with two unimpaired participants. Transparency was quantified by the magnitude of unwanted robot interaction forces. In a small six-session feasibility study, six individuals who had prior strokes telerobotically interacted with two trainers (separately), who assisted in altering a targeted gait feature: an increase in the affected leg’s swing length.

**Results:**

During unassisted walking, unwanted robot interaction forces averaged 3−4 N (swing–stance) for unimpaired individuals and 2−3 N for the patients who survived strokes. Transients averaging about 10 N were sometimes present at heel-strike/toe-off. For five of six patients, these forces increased with treadmill speed during stance (R^2^ = .99; p < 0.001) and increased with patient height during swing (R^2^ = .71; p = 0.073). During assisted walking, the trainers applied 3.0 ± 2.8 N (mean ± standard deviation across patients) and 14.1 ± 3.4 N of force anteriorly and upwards, respectively. The patients exhibited a 20 ± 21% increase in unassisted swing length between Days 1−6 (p = 0.058).

**Conclusions:**

The results support the feasibility of locomotor assistance with a telerobotics approach. Simultaneous measurement of trainer manipulative actions, patient motor responses, and the forces associated with these interactions may prove useful for testing sensorimotor rehabilitation hypotheses. Further research with clinicians as operators and randomized controlled trials are needed before conclusions regarding efficacy can be made.

## Background

Locomotor impairments can arise from injuries or disease processes that disrupt sensorimotor operations, such as spinal cord injuries and stroke. Locomotor training may be incorporated into a rehabilitation program. One approach uses a treadmill because it allows tight control over walking speed and terrain and facilitates the use of a body-weight support system [1, 2]. During treadmill training, human trainers can provide physical assistance to facilitate limb movement and support trunk stabilization [3]. High-volumes of task-orientated practice can promote neuroplasticity [4]. Although treadmill training has shown positive results for patient populations, including individuals who have had strokes [5, 6] or incomplete spinal cord injuries [7, 8], the overall efficacy is not unambiguously superior to other methods such as over-ground training or general exercise regimens [2, 9–13]. Explaining the inability of manual treadmill training to consistently meet expectations presents a grand challenge due to high investigational variability (e.g., eligibility criteria and intervention parameters [14]).

When providing physical assistance to elicit targeted modifications of a patient’s locomotor pattern, human trainers must contend with relatively complex patient dynamics. This includes the gravitational and inertial forces associated with the large wobbling mass [15] of a patient’s upper body, which is alternately supported by multi-link segmental chains (the legs) during locomotion. Further, the joints spanning these segments are actuated by a redundant set of viscoelastic muscles [16] controlled by a possibly impaired nervous system. Trainers also face significant sensorimotor constraints. They often need to produce large forces while kneeling or sitting with a limited view of a patient’s body and need to keep up with rapidly swinging patient limbs to prevent unintended interaction forces, which demands predictive control processes due to sensorimotor delays [17]. The combination of complex interactive dynamics, high forces, and rapid movements creates a challenging task that may limit trainer effectiveness.

One way to address the physical limitations of human trainers is to enlist the help of robots [18–20]. However, in doing so, human trainers have been relegated to a supervisory role. At the same time, robotic gait training outcomes have not proven dependably better than conventional rehabilitation approaches for spinal cord injury [21, 22] or stroke [23–27]. Giving trainers more online control of the robotic system (trainer-in-the-loop) could improve rehabilitation outcomes. The rationale is that trainers can use their experience and judgment to customize locomotor assistance on the fly, and their relatively high degree of motor execution variability could be a feature instead of a bug. Self-generated (intrinsic) variability can promote the exploration of novel motor actions that drive learning [28, 29]. Could this also hold for variability injected from an external source, i.e., from a trainer to a patient? If so, these advantages could be masked by trainer fatigue or other sensorimotor encumbrances. These points lead to the principal question: Would treadmill training outcomes improve if trainers remained in control, were relieved of high physical demands, and received augmented feedback about their patient interactions? Telerobotics, or robotics with a human operator in the control loop [30], may provide a viable approach to answering this question.

Although telerobotics has been broadly researched, for example, in areas related to telesurgery (see [30] for a review), rehabilitation applications with continuous physical interaction between clinicians and patients are more limited. Most existing telehealth approaches only permit visual and auditory communication [31–34]. One study investigated the feasibility of remote haptic communication using an exoskeleton to record the movements of patients who have had strokes; these movements were subsequently played back using an exoskeleton worn by therapists [35]. By feeling the patients' movements through the exoskeleton, the therapists were able to identify abnormal movement patterns. Although the results are promising, the therapists did not command the robotic system to apply forces to patients. Others have recently explored impedance-based telerobotics approaches for upper-extremity rehabilitation [36, 37], but such techniques have yet to be tested in clinical populations. In general, there is a significant need for telerobotics approaches that allow real-time bidirectional physical interaction between trainers and patients [38], which, in addition to benefits associated with human–human interaction (see previous paragraph) may be useful with heightened disease transmission risks.

### Structure of report

This report is divided into three sections:

1. The first section introduces a telerobotics approach that allows online physical interaction between a trainer and patient. Here, the approach is embodied in the context of locomotor recovery. A trainer holds onto a small robotic manipulandum that provides real-time haptic feedback about a patient’s locomotor actions, so the trainer feels like they are holding onto a miniature version of the patient (Fig. 1a). A visual display shows information related to patient errors and training progress. The trainer can assist by applying light forces to the manipulandum, which are amplified and transferred to the patient’s affected leg using a magnetically-attached robotic arm (i.e., teleoperation). The use of a robotic arm for locomotor training deviates from conventional approaches that use exoskeletons [39–42], cables [43, 44], or footplates [45, 46] to apply force to patients. In contrast to an exoskeleton, a magnetically-attached robotic arm requires only a small piece of metal embedded in a brace or garment to engage with a patient. With an array of suitable attachments, a robotic arm can interface with most body parts.

**Fig. 1:**
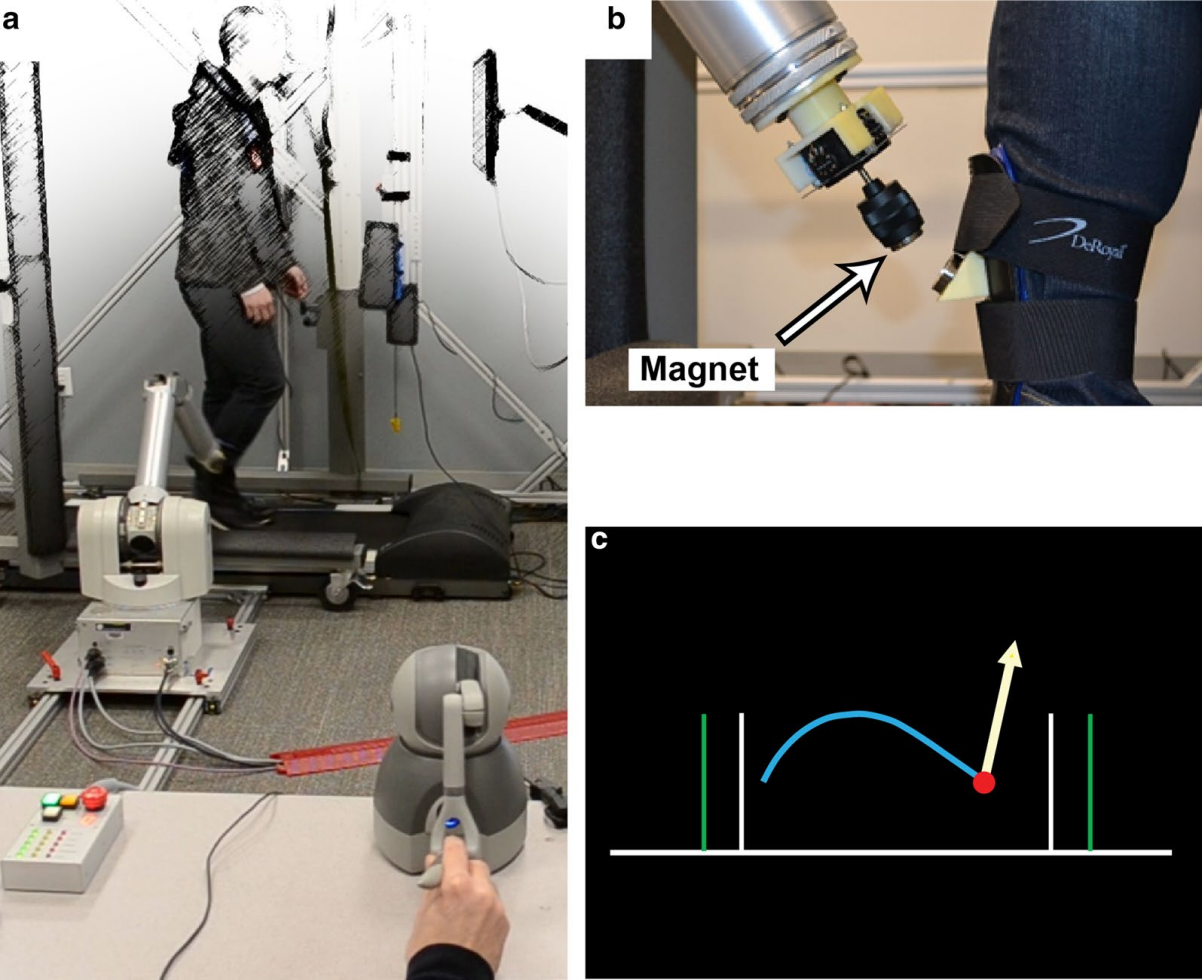
Overview of telerobotics locomotor rehabilitation approach. **a** A patient walks on a treadmill with a large robot arm connected to their right lower leg using a magnetic attachment. Meanwhile, a human trainer holds a small robot manipulandum, which shadows the scaled-down motion of the large robot arm and allows the trainer to feel and manipulate the patient’s gait in real-time (i.e., teleoperation). **b** A close-up view of the magnetic attachment (detached for clarity). **c** A visual display is provided to the trainer, which shows the patient’s gait kinematics and task-related overlays (e.g., gait target, force magnitudes, etc.). The white and green vertical lines show the actual and target swing lengths, respectively. The white arrow shows the trainer’s applied force
2. The second section of this paper describes the results of preliminary tests on unimpaired participants. These tests focused on the system’s transparency, which refers to the relative ease at which an individual can move (or back-drive) the robotic arm during unassisted locomotion, which constitutes a zero-impedance, zero-force, or patient-in-charge mode of robot operation [47]. This section also validates a procedure that reduces errors in swing-length estimation due to small day-to-day variations in the location of the robot attachment on the patient’s leg.
3. The third section describes a small feasibility study with six individuals who have experienced strokes. The purpose of the feasibility study was to gather information to design more extensive clinical trials that answer the main question posed earlier: whether the outcomes of treadmill training would improve if human trainers remained in control, were relieved of high physical demands, and received augmented feedback about their patient interactions. Several preliminary questions were formulated:
  *Regarding transparency:* In a small clinical sample constituting individuals who have had prior strokes, how transparent is the telerobotic system during locomotion? How is the system’s transparency affected by treadmill speed and patient anthropometry? (n.b., the magnitude of unwanted interaction forces quantified transparency.)
  *Regarding the trainers:* What forces do trainers command with the small manipulandum when providing locomotor assistance to patients? How do trainer manipulandum forces change from day-to-day; how do they differ between trainers? To what degree do the forces applied to patients by the robotic arm reflect those commanded by trainers?
  *Regarding the patients:* Can the patients complete a 6-day telerobotics locomotor training protocol successfully? Is the telerobotics system able to elicit a targeted change in patient gait, operationally defined as a 25% increase in the affected leg's swing length? Do these changes persist over multiple training sessions?
    The clinical population was chosen because hemiplegia is typically associated with an asymmetric locomotor pattern, such that one leg has a shortened step length and may have difficulty with ground clearance [48]. In this case, only a single robotic arm is needed to assist the affected leg. Future work can extend the approach to other populations, for example, by adding a second robotic device or working collaboratively with human trainers. It should be emphasized that rather than providing definitive answers, this study was designed to provide preliminary data related to each of the questions posed, to be evaluated further in future studies.

## Methods

### Section 1: Description of the telerobotics approach

#### Robot-patient coupling

Locomotor assistance was provided with a commercially available robotic arm (WAM; Whole-Arm Manipulator, Barrett Technology, Newton MA), which was magnetically attached to the lower leg near its center of mass (Fig. 1a). The robot-patient coupling consisted of two parts: a semi-rigid ankle brace with a small steel cup worn by patients and a joystick and magnet mounted on the end of the robot (Fig. 1b). This coupling improves upon an earlier design [49] by adding the joystick (M31L081P, APEM Inc.), which has potentiometers to measure the coupling angle along two axes; the third axis (axial rotation) was not measured. The joystick added three passive degrees of freedom (DOFs) to the robot arm, bringing the total DOFs to seven (four active and three passive; the four active DOFs were measured with incremental encoders with a resolution of 0.005°). The additional passive DOFs provided sufficient robot mobility to limit undesired interaction torques between the robot and patients. The robot attachment state was monitored with a pair of DuPont connectors that pulled apart, opening a circuit if the robot detached from the leg. The joystick potentiometer and attachment state circuit voltages were digitized by a microcontroller (Pro Micro 5 V/16 MHz, SparkFun) and sent via USB to the robot control computer. A 3D-printed plastic housing held the joystick, which was bolted to a force sensor on the end of the robotic arm. The force sensor used bonded silicon strain gauges with a resolution (i.e., smallest distinguishable difference) of 50 mN perpendicular to the long axis of the end effector and 80 mN axially.

#### Mechanical fuse

A neodymium ring magnet (RX038DCB, K&J Magnetics Inc.) acted as a mechanical fuse to separate the robot from the patient in the event of a mechanical overload. The magnet was screwed into the joystick on the end of the robot (Fig. 1b). The magnet had an axial pulling force of 176 N. This magnet strength was chosen so that it would be strong enough to securely maintain the robot-human connection but would release if an unusual force or torque was applied (mechanical overload). This was determined empirically by the investigators, who separately walked on a treadmill with the robotic arm attached to their legs. Various magnet strengths were tested by walking at different speeds with different symmetrical and asymmetrical gaits; different stumbling actions were also reproduced (a safety harness was worn). The magnet strength was chosen so that the robotic arm stayed attached during all walking variations, including minor stumbles; however, when a stumble caused an exceptionally severe alteration in walking kinematics, there was a complete loss of balance, or if the tester stopped walking (and thus moved rearward), the robot detached (these were considered mechanical overloads).

#### Robotic arm control

The robotic arm used for the patient interface achieved high intrinsic back-drivability with a low-friction cable transmission system with zero-backlash, i.e., there was a tight linkage between the commanded motor torques and the resulting torques on the joints of the robot arm. A dedicated real-time computer (Ubuntu 14.04.5; Linux Kernel 3.14.17; Xenomai 2.6.4) closed a 1000 Hz torque control loop using a CAN (Controller Area Network) bus for rapid real-time communication between the computer and the robotic arm. The control loop incorporated a series of actions: sending position information to the computer, calculating desired torques, and sending appropriate motor torque commands to the robotic arm. The torque controller included gravity compensation to support the robot arm’s weight. For this compensation, a manufacturer-supplied routine estimated the first moment of mass vectors for each robot linkage (assuming a static case; for more details, see manuals and documentation provided by the manufacturer, Barrett Technology). Henceforth, the manufacturer-supplied torque controller is referred to as the native controller.

Typical rehabilitative applications of robot arms include goal-directed upper-extremity tasks [50–52]. In this study, the robotic arm was attached to the lower-leg during locomotor training, which places a unique set of demands on the robot controller. The human gait cycle typically includes a brisk low-impedance swing phase, followed by a large impact at heel-strike, and a slower high-impedance stance phase. High back-drivability of the robot arm during the swing phase was of particular concern. Pilot testing showed that although the native controller permitted a good deal of back-drivability, the robot arm's presence was noticeable during the swing phase of gait (the native controller does not compensate for the robot’s inertia and other unwanted forces). Therefore, this study sought to improve the transparency of the native controller, i.e., increase the ease at which the patient’s leg can back-drive the robot and reduce unwanted interaction forces (forces other than those applied by a human trainer operating the system).

**Fig. 2:**
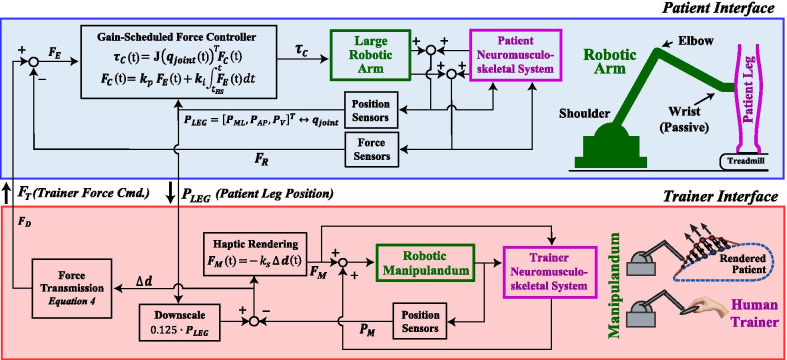
Flowchart detailing operation of the telerobotics system. A large robotic arm is magnetically attached to a patient’s leg. The motion of the patient’s leg is measured using the robotic arm’s sensors. A scaled-down version of the patient’s motion is haptically rendered using a small robotic manipulandum, which is held by a human trainer. The trainer can provide assistance (or resistance) in real-time by deflecting the manipulandum at an appropriate point in the gait cycle; the amount of force transmitted is proportional to the deflection (within limits)

One approach to improving transparency is to incorporate information about end-effector interaction forces into the control scheme, which can be used to compensate for unmodeled robot arm dynamics, such as inertia [39, 53–55]. The force sensor mounted on the end of the robotic arm measured the three-dimensional force applied to the patient ${\varvec{F}}_{{\varvec{R}}}$ (boldface denotes a vector quantity). A gain-scheduled proportional-integral force controller (Fig. 2) was used to improve transparency and transmit assistive or resistive forces commanded by a human trainer ${\varvec{F}}_{{\varvec{T}}}$. For unassisted/unimpeded patient locomotion ${\varvec{F}}_{{\varvec{T}}} = 0$; more details on determining ${\varvec{F}}_{{\varvec{T}}}$ are presented in the *Trainer Interface* section that follows. To render ${\varvec{F}}_{{\varvec{T}}}$ on the patient, the controller regulated the robot arm motor torques ${\varvec{\tau}}_{{\varvec{C}}}$ (three shoulder/one elbow) according to

1 $${\varvec{\tau}}_{{\varvec{C}}} \left( t \right) = {\text{J}}_{ } \left( {{\varvec{q}}_{{{\varvec{joint}}}} \left( t \right)} \right)^{{\text{T}}} {\varvec{F}}_{{\varvec{C}}} \left( t \right)$$

where ${\text{J}}_{ } \left( {{\varvec{q}}_{{{\varvec{joint}}}} } \right)^{T}$ is the Jacobian transpose matrix (transforms endpoint forces to joint torques), ${\varvec{q}}_{{{\varvec{joint}}}}$ are the robot arm joint angles, and ${\varvec{F}}_{{\varvec{C}}}$ is the controller force defined as

2 $${\varvec{F}}_{\varvec{C}} \left( t \right) = k_{p} {\varvec{F}}_{{\varvec{E}}} \left( t \right) + k_{i} \mathop \smallint \limits_{{t_{HS} }}^{t} {\varvec{F}}_{{\varvec{E}}} \left( t \right)dt,$$

where $$k_{p}$$ and $$k_{i}$$ are proportional and integral gains indexed to locomotor events, $${\varvec{F}}_{{\varvec{E}}}$$ is the force error ($${\varvec{F}}_{{\varvec{E}}} = {\varvec{F}}_{{\varvec{T}}} - {\varvec{F}}_{{\varvec{R}}}$$), $$t_{HS}$$ is a timestamp marking the previous heel-strike, and $$t$$ is a timestamp identifying the most recent force and position measurements (updated every millisecond). The timestamp $$t_{HS}$$ was identified online as the maximum anterior displacement of the robot arm end-effector on the previous step; this event typically occurred just before the heel contacted the treadmill. The integrated error was reset to zero after each step at $$t_{HS}$$. Under this control scheme, the higher the gains ($$k_{p}$$ and $$k_{i} )$$, the more aggressively the robot arm tried to drive $${\varvec{F}}_{{\varvec{E}}}$$ to zero.

A gain-scheduled force controller was used because the mechanical impedance (a dynamic extension of stiffness [56]) of the human leg varies significantly across the different phases of gait [57]. When the robotic arm is connected to a low-impedance environment, such as the leg during the swing phase of gait, a high force controller gain is desirable as it makes the robot controller aggressively track ${\varvec{F}}_{{\varvec{T}}}$. However, when the robot is connected to a high-impedance environment, e.g., the leg during the stance phase, contact instabilities can result, particularly with high force-feedback gains [56]. Since one would typically not try to move an individual’s leg out from beneath them when it supports a portion of their body weight during stance, the controller gain can be lowered during this time. Specifically, just before heel-strike ($t_{HS}$) the controller gains were lowered to $k_{p} =$ 1.0 and $k_{i} =$ 0.005. The gains were held constant during the stance phase until just before heel-off; the latter was identified online as the maximum posterior displacement of the robot arm end-effector. Subsequently, the gains were raised to $k_{p} =$ 6.0 and $k_{i} =$ 0.4 during leg swing until the next $t_{HS}$. The gains were determined using a manual tuning process with unimpaired participants walking on a treadmill. The gains were initially set low and gradually increased until there was a noticeable oscillation of the robotic arm, which indicated that the controller’s stability was becoming compromised. Gain values just below the level of noticeable robot oscillation were chosen separately for the stance and swing phases of gait.

#### Trainer interface

Integral to the telerobotics approach is a trainer interface that consisted of a small robotic manipulandum (Geomagic Touch, 3D Systems, Rock Hill, SC) and visual display (Fig. 1c). The manipulandum followed a scaled-down version of the robotic arm end-effector position, shadowing the movement of a patient’s leg during treadmill locomotion (see flowchart in Fig. 2). This tracking was achieved using a virtual spring, which commanded the manipulandum motors to produce torques to render a manipulandum end-effector force ${\varvec{F}}_{{\varvec{M}}}$ according to

3 $${\varvec{F}}_{{\varvec{M}}} \left( t \right) = - k_{s} \Delta {\varvec{d}}\left( t \right)$$

where $\Delta {\varvec{d}}$ is the difference between the measured manipulandum position and the scaled robot arm end-effector position ${\varvec{P}}_{{{\varvec{LEG}}}}$, and $k_{s}$ is a spring constant (0.2 N/mm). ${\varvec{P}}_{{{\varvec{LEG}}}}$ was downscaled by a factor of 0.125. The constant $k_{s}$ was determined by asking a human trainer to hold onto the manipulandum as another healthy individual walked on a treadmill with the robotic arm attached to their leg. The human trainer was instructed to just follow along and not interfere as $k_{s}$ was varied. The $k_{s}$ used was the highest that permitted good tracking performance without noticeable oscillations.

**Fig. 3:**
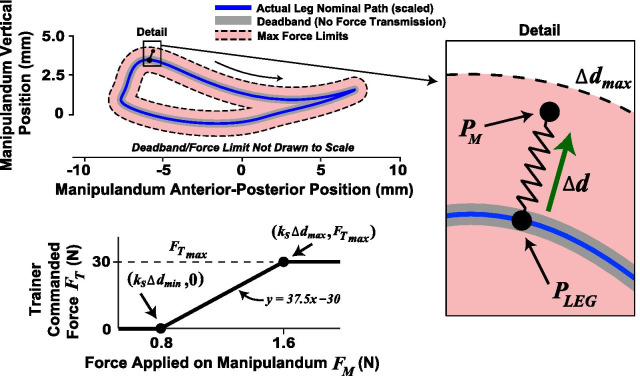
Trainer force transmission algorithm. A trainer can apply force to a patient by deflecting a manipulandum, which follows a scaled-down version of the patient’s leg motion in real-time. The trainer’s force is amplified and scales with the amount of deflection. Note the gray shading showing the deadband and the red shading showing the range of transmitted forces are not drawn to scale

The manipulandum control scheme was modified so that, instead of just following along, the human trainer could dynamically take charge and deliver a time-varying manipulative force to a patient. The goal was to use an intuitive approach. For example, if a trainer wants to assist with foot clearance, the trainer should be able to lift and deflect the manipulandum upwards at the appropriate time in the gait cycle. Further, the more the trainer pushes on the manipulandum, the more the manipulandum should deflect, and the force transmitted to the patient should increase (within limits). This deflection is captured by $\Delta {\varvec{d}}$. The force transmission scheme (Fig. 3) was implemented as (Eq. 4)

**Figure:**
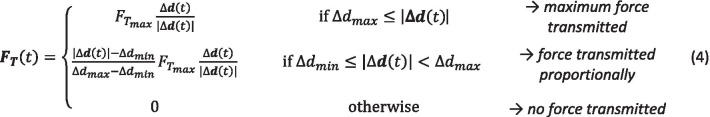

where ${\varvec{F}}_{{\varvec{T}}}$ represents the trainer’s commanded force (which the robot arm tries to produce; see Eq. 2 and accompanying text), the threshold $\Delta d_{min}$ (4 mm) defined the radius of a deadband about the downscaled patient leg position, and $\Delta d_{max }$ (8 mm) scaled the overall amount of force amplification by establishing the maximum desired assistance force ($F_{{T_{max} }} = 30{\text{N}}$). If $\left| {\Delta {\varvec{d}}} \right|$ was below $\Delta d_{min}$ then no force was commanded. If the trainer force exceeded the deadband, then a force was commanded that scaled with $\Delta {\varvec{d}}$. The deadband was necessary so that sensorimotor noise in the trainer did not cause unintended force commands. The effective amplification factor increased with the trainer input force, with an amplification factor of 18.75 at $\Delta d_{max }$. $F_{{T_{max} }}$ was based on the maximum payload of the robotic arm (about 40 N), reduced by a safety factor of about 35%. Note that the direction of ${\varvec{F}}_{{\varvec{T}}}$ was always the same as $\Delta {\varvec{d}}$ and force transmission occurred in two-dimensions aligned with the sagittal plane of the patient. The trainer forces were software limited to be in the anterior and upwards directions so that the trainer could not command forces that resisted patient motion. This limiter could be turned off in software and was in one case for Patient 3. No force was transmitted during a time window starting at heel-strike and ending 200 ms later.

#### Trainer visual display

During locomotor training, trainers received visual feedback on a computer monitor. The visual display showed the location of the robot arm endpoint (attached to a patient’s leg) and was updated at 50 Hz (Fig. 1c). A short “tail” was provided, showing the last second of motion. A target swing length was shown as two vertical lines. Although the orientation of the patient’s leg could be estimated and shown to the trainer on the visual display, a recent study by Hasson and Jalili [58] showed that seeing the patient’s leg does not provide a significant benefit in terms of learning the patient dynamics, and therefore only the robot attachment point was shown.

### Section 2: Preliminary work

#### Robot arm controller transparency

Given that transparency (i.e., the ability of the robotic system to move with a patient during unassisted locomotion) is a basic requirement of most robotic rehabilitation systems, an initial transparency evaluation was performed with two unimpaired male participants (U1: investigator MK; U2: investigator CJH; see Table 1 for descriptive characteristics). The participants walked on a treadmill at 0.45 m/s with and without the robot attached to the leg. At this very early stage of evaluation, the investigators participated so that any necessary refinements could be made prior to recruiting naive individuals or patients (all procedures were approved by the Northeastern University and Tufts Medical Center Institutional Review Boards). In each condition, the participants walked for four minutes while leg kinematics were collected using a four-camera optical motion-capture system (120 fps, Flex 13, OptiTrack, NaturalPoint, Inc., Corvallis, OR). Passive reflective markers (1 cm spheres) were placed on the right leg at two locations: one representing the hip joint center (greater trochanter of the femur) and one representing the robot attachment site (the proximal aspect of the rim of the metal cup accepting the magnetic attachment from the large robotic arm). Markers were tracked in 3D (Motive version 1.9.0, NaturalPoint, Inc., Corvallis, OR) and the 2D sagittal plane coordinates were exported to MATLAB for analysis. Only the second half of each four-minute walking trial was analyzed. The motion of the patient’s leg $({\varvec{P}}_{{{\varvec{LEG}}}}$) and the force exerted on the participants from the robotic arm (${\varvec{F}}_{{\varvec{R}}}$) were filtered with a 4th order zero-lag low-pass Butterworth filter with a cut-off frequency of 20 Hz.

**Table 1:** Characteristics of unimpaired participants used in the preliminary transparency evaluation (U1 & U2) and validation procedure for a virtual robot attachment site (U3–U6)

Measure	U1	U2	U3	U4	U5	U6
Age (decade)	30 s	30 s	20 s	20 s	20 s	20 s
Height (m)	1.73	1.88	1.88	1.70	1.77	1.63
Weight (kg)	85	79	79	64	66	64
Sex	Male	Male	Male	Male	Female	Female

#### Swing length estimation

The leg swing length was used in the patient feasibility study. This variable is determined from the peak-to-peak displacement of the robotic arm end-effector position, which was near the shank center of mass at position ${\varvec{P}}_{{{\varvec{LEG}}}}$. The term swing length is used because step length, a common measure of gait, is typically defined using displacements of landmarks on the foot [59, 60], which was not tracked in this study. This attachment location may exhibit small variations from day-to-day, which will affect the estimated swing length and height. For example, shifting ${\varvec{P}}_{{{\varvec{LEG}}}}$ distally by 2% of the shank length $l_{s}$ for a 170 cm tall individual increases swing length by about 1 cm (based on a two-link locomotor model; see below). Thus, swing length and height were estimated from a virtual robot attachment site, denoted as $\hat{\user2{P}}_{{{\varvec{LEG}}}}$, which could be placed at the exact same percentage of $l_{s}$ on each day (the mean across practice days for a given participant). To determine $\hat{\user2{P}}_{{{\varvec{LEG}}}}$ the shank orientation was estimated using a simple two-link rigid body model of the participants’ affected leg. This model estimated the thigh and shank lengths based on participant height and regression equations in Winter [61] and assumed a quasi-static hip joint at a vertical distance equal to the total leg length, centered over the swinging leg to account for drift across the treadmill. The model assumed planar motion and hinge-like hip and knee joints.

To assess the validity of the model-based robot attachment site adjustment procedure, three-dimensional lower-extremity locomotor kinematics were measured for two unimpaired male adults and two unimpaired female adults (U3–U6; Table 1) during treadmill walking. Unimpaired participants were used because the validation procedure required accurate measurements of the shank displacement and orientation, but the data collected on patients only measured the linear displacement of the point of attachment using the robotic arm sensors. For U3–U6, the position and orientation of the shank were measured from retroreflective spherical markers placed on the fibular apex of the lateral malleolus and lateral femoral epicondyle. Next, a single point on the shank was selected at the mean location of $${\varvec{P}}_{{{\varvec{LEG}}}}$$ for the patient data (39.1% of $$l_{s}$$). This point was shifted either proximally or distally by an amount that reflected the natural variation in $${\varvec{P}}_{{{\varvec{LEG}}}}$$ for the patients (two standard deviations or about 2% of $$l_{s} )$$. Finally, the swing length was calculated using $$\hat{\user2{P}}_{{{\varvec{LEG}}}}$$. The root-mean-squared error between the estimated and true (motion-capture-based) swing length was 0.16 ± 0.03 mm for a two-standard deviation proximal shift of 8.4 mm and 0.21 ± 0.03 mm for a distal shift of the same magnitude. Given that the observed changes in swing length for the patients were an order of magnitude higher, any errors in swing length estimation would have a negligible impact on interpreting the results for the patient data.

### Section 3: Locomotor training with individuals who had prior strokes

#### Experimental design and participants

A small study was performed to explore the feasibility of the telerobotics approach with six individuals who have experienced strokes (Table 2). The inclusion criteria were: adults older than 18 years of age, prior history of acute ischemic stroke or intracerebral hemorrhage, beyond 90 days since stroke onset, established neurological deficit of hemiparesis affecting the leg, able to ambulate on a treadmill without another person assisting, able to consent by themselves or by proxy in English. The exclusion criteria were: recent symptoms (within 7 days) of chest pain, any medical condition that creates undue risk related to the exertional demands of the study, or if they were unable to consent. All patients provided written informed consent, and the Institutional Review Boards of Northeastern University and Tufts Medical Center approved the experimental procedures.

**Table 2:** Characteristics of study participants who had previously experienced a stroke

Measure	P1	P2	P3	P4	P5	P6
Age (decade)	50 s	20 s	30 s	60 s	60 s	40 s
Height (m)	1.78	1.57	1.73	1.78	1.60	1.70
Weight (kg)	104	72	140	104	73	63
Sex	Male	Female	Male	Male	Male	Male
Years Since Stroke	16	1	3	4	1.5	1
Affected Lower Extremity	Left	Left	Left	Left	Right	Right
Overground Speed (m/s)^a^	1.19	N/A^b^	0.48	N/A^b^	N/A^b^	N/A^b^
Training Treadmill Speed (m/s)^a^	0.54	0.18^c^	0.45	0.27	0.18^c^	0.27
Assistive Device for Ambulation	None	Walker	Single Cane	Walker	Wheelchair	Wheelchair
Functional Independence Measure Rating^d^	7: Complete Independence	4: Contact Guarding	6: Modified Independence	3: Moderate Assistance	1: Total Assistance	1: Total Assistance
Location and Type of Stroke	Right Thalamic Intracerebral Hemorrhage	Right Pontine Ischemic Stroke	Spinal Cord Ischemic Stroke	Right Basal Ganglia, Right Frontal Lobe; Ischemic Stroke	Left Frontal Lobe, Left Parietal Lobe; Ischemic Stroke	Left Frontal Lobe, Left Parietal Lobe; Ischemic Stroke

^a^Based on Perry et al. [69]: household ambulation = overground walking speed < 0.4 m/s; limited community ambulation = 0.4 to 0.8 m/s; full community ambulation > 0.8 m/s

^b^GaitRite was used to measure walking speed; thus, measurements could not be taken if an assistive device was require

^c^Slowest possible treadmill setting

^d^Based on Fiedler et al. [70]

This feasibility study focused on the basic question of whether the telerobotics approach can elicit a change in a targeted gait feature. In all patients, there was a gait asymmetry, such that one leg had a shorter leg swing (physician indicated and supported by observations); thus, an increase in swing length was used as the training goal (specifically, a 25% increase in swing length for the paretic leg). Swing length was defined as the peak-to-peak anterior–posterior displacement of the mid-shank (location of robotic arm attachment). The rationale was that an increase in paretic leg swing (step) length typically improves locomotor symmetry, which may, in turn, reduce energy expenditure [62, 63] and positively impact the musculoskeletal health of the non-paretic limb [48]. However, note that an increase in paretic swing length will not always translate into a reduction in gait asymmetry [64], and some individuals may naturally take longer steps with the paretic leg [65–67]. A scalar gait metric (swing length) was chosen as the goal instead of a target kinematic pattern because it was expected to be simpler for the patient and trainer to cooperatively achieve in real-time. Additionally, not constraining movement trajectories to a specific pattern allows more joint-level variability, which may promote sensorimotor recovery [68].

#### Experimental procedures

All training was performed in a dedicated hospital room at Tufts Medical Center. Each patient received physical assistance from human trainers using the telerobotics approach while walking on a treadmill (TR500B, Life Fitness, Rosemont, Illinois) on six separate days. On each day of training, patients were required to have an asymptomatic, pre-exercise systolic blood pressure less than 160 mmHg, diastolic blood pressure less than 100 mmHg, and resting heart rate under 150 bpm. Before commencing with locomotor training on the first day, a familiarization trial was performed without the robot attached. The treadmill speed was adjusted until the patient reached a walking speed that resulted in a self-described exertion level between “somewhat hard” and “hard (heavy)” on the Borg Rating of Perceived Exertion (RPE) scale [71]. This trial lasted from about 30 s to a maximum of 5 min, depending on the patient’s exertional capacity (some patients grew tired quickly). This treadmill speed (Table 2) was used for subsequent training sessions. Next, the patients received assistance through the telerobotics system. The trainers placed the magnetic attachment brace on the patient’s shank close to the center-of-mass using standard anthropometric criteria as a guideline (e.g., shank COM is about 43% of the distance from the femoral condyle to the lateral malleolus; see [61]). The first 5–20 steps of each trial were unassisted, followed by assistance from a trainer via the telerobotics system (more heavily impaired individuals needed help sooner to prevent undue fatigue). The kinematics associated with the initial unassisted steps on Day 1 were used to calculate the target swing length (a 25% increase), which remained the same throughout training (except for P1, for whom the target was increased on Day 2 to maintain the task challenge).

The number and duration of telerobotics assistance trials varied according to each stroke survivor’s exertional capacity and how they felt during the day of training. Patients performed between 2 and 5 trials/day averaging 4.9 ± 1.8 min (mean ± standard deviation across patients) for each trial. The maximum duration for a single walking trial was limited to 10 min. During training, patients were instructed to avoid using compensatory strategies such as hip-hiking or circumduction. If such strategies were observed, a physical therapist (investigator SCY) provided instructions to help the patient avoid relying on a compensatory strategy, to the extent possible. The physical therapist monitored the patients at all times during treadmill locomotion. After each trial, patients were asked to indicate their RPE on a visual chart, and heart rate and blood pressure measurements were taken. Periodic verbal checks of patient status were made during each trial; any cases of persistent lightheadedness, chest pain, headache, or new/worsening stroke symptoms prompted exercise termination and clinical assessment (no such terminations were necessary).

For this feasibility study, two of the investigators (Trainer 1 = CJH; Trainer 2 = MK) took on the role of the trainers who operated the manipulandum. Both trainers had several hours of experience operating the telerobotics system but were not clinicians. The justification was threefold: this was the first feasibility study performed that focused on the patient experience, training protocols for clinicians using the system do not exist, and resources were not available to hire additional physical therapist(s) to perform the training for the 36 sessions.

For the first three stroke survivors (P1–P3), Trainer 1 provided assistance for all training days except the fifth day on which Trainer 2 assisted. This switch was initially performed out of necessity: during the fifth training session for P1, Trainer 1 was unable to participate. This was used as an early opportunity to explore how a different trainer might operate the system, and therefore the same switch was implemented for P2 and P3 on the fifth training day. To further examine differences between the trainers, Trainer 2 provided assistance using the telerobotics system for all six sessions for patients P4–P6. The patients wore a safety harness (Unweighting System 945–480, Biodex Medical Systems, Inc., Shirley, NY), but no unweighting was applied, and they were permitted to hold onto supporting rails if needed (otherwise, training time would be very short due to patient fatigue [72]).

#### Data analysis

*Transparency:* The transparency of the telerobotics system with individuals who survived strokes was evaluated by calculating the average resultant force magnitude (across all practice days) exerted on each patient by the robotic arm (${\varvec{F}}_{{\varvec{R}}}$) during the initial no-assistance steps. The average maximum ${\varvec{F}}_{{\varvec{R}}}$ was used to assess the worst-case transparency of the telerobotics system (typically, at heel-strike).

*Factors affecting transparency:* Pearson correlations were performed to identify patient characteristics that might explain the variance in the degree of transparency. During the stance phase, the leg is constrained by the treadmill; thus, the correlation between treadmill speed and the average ${\varvec{F}}_{{\varvec{R}}}$ during stance was determined. Based on the pendular action of the leg during leg swing, taller patients would be expected to have faster leg swings (notwithstanding gait abnormalities), which could increase the challenge for the robot controller. On the other hand, low velocities could also create control challenges due to stick–slip phenomena [73]. To explore these possibilities, the correlation between patient height and swing-phase ${\varvec{F}}_{{\varvec{R}}}$, and the correlation between the maximum leg swing velocity and swing-phase ${\varvec{F}}_{{\varvec{R}}}$, were also determined.

*System performance with augmented trainer assistance:* The correspondence between the forces commanded by the trainers (${\varvec{F}}_{{\varvec{T}}}$) and the forces applied by the robotic system on patients’ legs (${\varvec{F}}_{{\varvec{R}}}$) was quantified by the root-mean-squared error between the magnitude of ${\varvec{F}}_{{\varvec{T}}}$ and ${\varvec{F}}_{{\varvec{R}}}$. This error was calculated for the entire swing phase (from heel-off to heel-strike) and just the late swing phase (toe-off to heel-strike).

*Patient adaptation and retention:* Measures of swing length and height were used to determine whether the telerobotics training elicited changes in unassisted patient locomotor patterns. The swing length was the difference between the peak rearward and peak anterior $\hat{\user2{P}}_{{{\varvec{LEG}}}}$. The swing height was the difference between the minimum and maximum $\hat{\user2{P}}_{{{\varvec{LEG}}}}$ along the vertical axis. The average unassisted swing length on the first trial of training Day 1 was compared with the unassisted swing length on the first trial of training Day 6 to quantify retention. The greatest daily change in unassisted swing length, relative to Day 1, was also calculated. In the course of training, there were occasional outlier steps due to events such as small stumbles, momentary losses of balance, and the foot hitting the ground prematurely. These outlier steps were defined as those with step lengths outside a 95% confidence interval for a trial (± 1.96 standard deviations) and were removed from further analysis.

#### Statistics

Permutation tests were used to evaluate differences among sample means [74]. Such tests were chosen as they are applicable to small samples and do not require distributional assumptions [75]. All data processing and statistical analyses were performed in MATLAB (R2020a, MathWorks Inc., Natick MA). One-sample permutation tests were used to test for significant differences from zero for the interaction force ${\varvec{F}}_{{\varvec{R}}}$, the root-mean-squared tracking error ${\varvec{F}}_{{\varvec{R}}} - {\varvec{F}}_{{\varvec{T}}}$, and changes in step length and height relative to Day 1. For each permutation test, the number of simulations was set at 5000 based on Marozzi [76]. The p-value was calculated as the fraction of permuted data that was larger than the sample mean $\overline{x}$ or smaller than $- \left| {\overline{x}} \right|$. Exact p-values are reported.

## Results

### Preliminary work

**Fig. 4:**
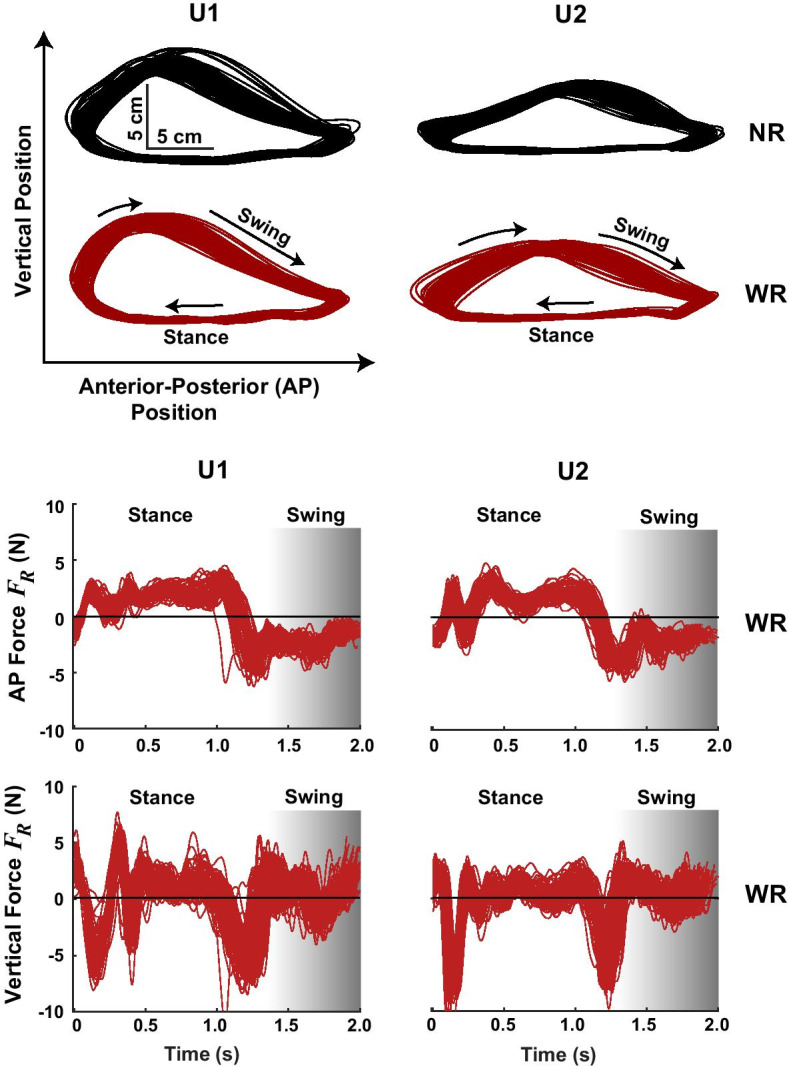
Evaluation of transparency in unimpaired participants: raw data. Trajectories and interaction forces for the right lower leg for two unimpaired participants (U1 & U2) during treadmill locomotion with and without a robotic arm attached to the leg. In a control condition (no robot; NR), the subjects walked without the robot attached to their legs (top; black trajectories). In the other condition (red trajectories), a robotic arm was attached close to the center-of-mass of the leg (with robot; WR), and the robot was programmed to follow the leg and not interfere. The measured interaction forces (${\varvec{F}}_{{\varvec{R}}}$) are shown from the reference frame of the subjects. A positive anterior–posterior (AP) force means the robotic arm was pushing anteriorly on the leg; a positive vertical force means that the robotic arm was pushing upwards on the leg. Force data are not interpolated to the step-cycle; therefore, the timing of the swing cycle varies slightly from step-to-step (hence, the gradient shading)

**Fig. 5:**
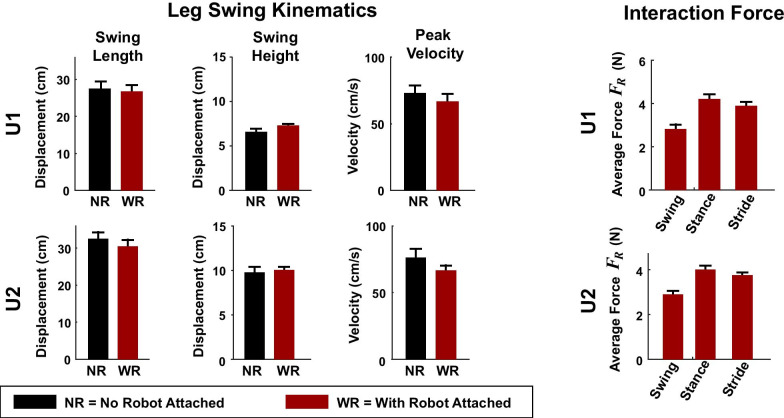
Evaluation of transparency in unimpaired participants: summary statistics. Step kinematics and robot interaction forces (${\varvec{F}}_{{\varvec{R}}}$) while two unimpaired subjects (U1 & U2) walked on a treadmill with (with robot; WR; red) and without (no robot; WR; black) a robotic arm attached to their legs. When the robotic arm was attached, it was programmed to follow along and not interfere with the subjects’ locomotion. The error bars denote one standard deviation across steps for the last two minutes of walking

Before performing the feasibility study with patients, the transparency of the robotic system was evaluated on two unimpaired individuals. Compared to walking without the robotic arm attached to their legs, the presence of the robotic arm was associated with a decrease in swing length of 4.5 ± 2.3% (mean ± standard deviation across subjects), an increase in swing height of 5.6 ± 5.3%, and a decrease in the peak swing velocity of 10.5 ± 2.8% (see trajectories in Fig. 4 and summary measures in Fig. 5). The interaction force ${\varvec{F}}_{{\varvec{R}}}$ with the robot attached to the leg averaged 4.1 ± 0.14 N during the stance phase and 2.85 ± 0.07 N during the swing phase (Fig. 5). There were brief periods of elevated vertical interaction forces at two time points: immediately post-heel-strike and at heel-off (Fig. 4, bottom). As the leg hit the ground, it accelerated upward, but the robot arm continued moving down and therefore applied a downward force to the leg. At heel-off, as the leg accelerated upwards, the robotic arm briefly lagged, similarly exerting a downward force on the subject’s leg. These lags were in part due to the lower gains used during the stance phase, which made the robotic arm controller behave less aggressively in its efforts to cancel unwanted interaction forces. Overall, this level of transparency was deemed suitable for further feasibility testing with patients.

### Locomotor training with individuals who had prior strokes

#### Transparency

For the patients, the average interaction force magnitude (${\varvec{F}}_{{\varvec{R}}}$) during the initial unassisted steps was 3.33 ± 0.60 N (mean ± standard deviation across patients) during the stance phase (different from zero, *p* = 0.029) and 2.25 ± 0.45 N during the swing phase (different from zero, *p* = 0.030). The average worst-case unassisted ${\varvec{F}}_{{\varvec{R}}}$ (average maximum across steps; typically occurring at heel-strike) was 10.28 ± 4.01 N during stance (different from zero, *p* = 0.032) and 5.05 ± 1.16 N during swing (different from zero, *p* = 0.036).

#### Factors affecting transparency

The correlation between the stance phase unassisted ${\varvec{F}}_{{\varvec{R}}}$ and treadmill speed was 0.78 (*p* = 0.067; R^2^ = 0.61); however, the correlation without P6 (who had the lowest interaction force) was 0.99 (*p* < 0.001; R^2^ = 0.99; $F_{R} = 2.8x + 2.6$ where $x$ is the treadmill speed in m/s). For the swing phase, the correlation between the unassisted ${\varvec{F}}_{{\varvec{R}}}$ and treadmill speed was 0.21 (*p* = 0.700; R^2^ = 0.04); without P6 the correlation was 0.15 (*p* = 0.816; R^2^ = 0.02). The correlation between swing-phase unassisted ${\varvec{F}}_{{\varvec{R}}}$ and patient height was − 0.26 (*p* = 0.617; R^2^ = 0.07); however, without P5 (the shortest patient) the correlation was 0.84 (*p* = 0.073; R^2^ = 0.71; $F_{R} = 9.7x - 15$, where $x$ is the patient height in meters) The correlation between the swing-phase unassisted ${\varvec{F}}_{{\varvec{R}}}$ and the maximum anterior-poster leg swing velocity was 0.36 (*p* = 0.480; R^2^ = 0.13); without P5 the correlation was 0.75 (*p* = 0.149; R^2^ = 0.55; $F_{R} = 1.4x + 1.1$ where $x$ is the maximum anterior–posterior leg swing velocity in m/s).

#### System performance with augmented trainer assistance

**Fig. 6:**
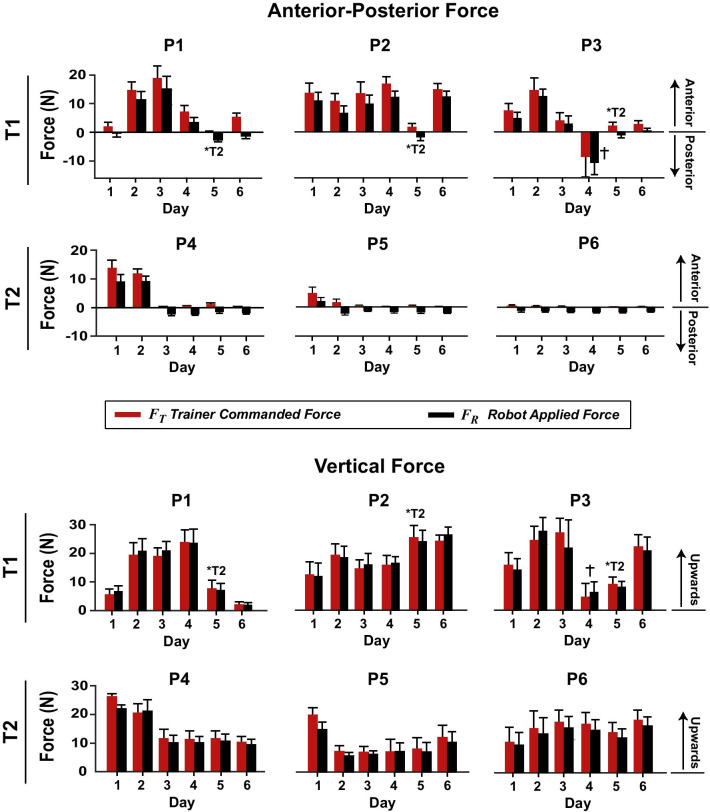
Trainer- and robotic arm-applied forces across 6 days of telerobotic locomotor training for six patients who have survived strokes (P1-P6). The *red* bars show the average commanded trainer force ${\varvec{F}}_{{\varvec{T}}}$ (average ± std. across steps) for the last trial of each day. This force is an amplified version of the force computed from the deflection of a manipulandum held by the trainer. The *black* bars show the average force applied to the patients’ affected legs by the robotic arm (${\varvec{F}}_{{\varvec{R}}}$). This force was measured by a force transducer on the end of the robotic arm close to where it was magnetically attached to the lower leg of the patients. P1-P3 received assistance from Trainer 1 (*except on Day 5 Trainer 2 assisted); P4-P6 received assistance from Trainer 2. ^†^P3 received resistance from Trainer 1 on Day 4. T1 = Trainer 1; T2 = Trainer 2

Across all with-assistance patient training sessions, during the phase from heel-off to heel-strike, the root-mean-squared tracking error between the measured and commanded trainer forces (${\varvec{F}}_{{\varvec{R}}} - {\varvec{F}}_{{\varvec{T}}}$) averaged 3.86 ± 1.27 N (mean ± standard deviation across patients) in the anterior–posterior direction (different from zero, *p* = 0.031) and 5.47 ± 1.38 N in the vertical direction (different from zero, *p* = 0.033). If only the late-swing phase is considered (from toe-off to heel-strike), the tracking error averaged 2.80 ± 0.71 N in the anterior–posterior direction (different from zero, *p* = 0.037) and 4.67 ± 1.02 N in the vertical direction (different from zero, *p* = 0.033). Typically, the anterior–posterior ${\varvec{F}}_{{\varvec{R}}}$ was less than ${\varvec{F}}_{{\varvec{T}}}$ with a negative (posterior) bias (Fig. 6). For the vertical component of ${\varvec{F}}_{{\varvec{R}}}$, a consistent bias was not observed across patients (e.g., for P1 ${\varvec{F}}_{{\varvec{R}}}$ was larger than ${\varvec{F}}_{{\varvec{T}}}$ and for P5 and P6 ${\varvec{F}}_{{\varvec{R}}}$ was smaller than ${\varvec{F}}_{{\varvec{T}}}$; Fig. 6). On average, the trainers applied 3.0 ± 2.8 N of force (${\varvec{F}}_{{\varvec{R}}}$) anteriorly and 14.1 ± 3.4 N of force (${\varvec{F}}_{{\varvec{R}}}$) upwards.

**Fig. 7:**
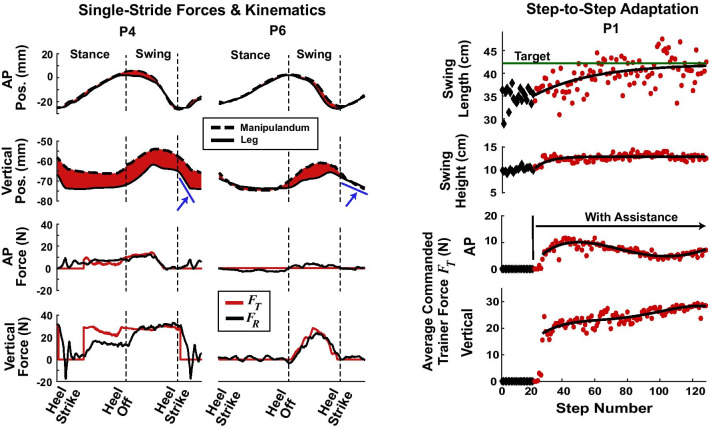
Examples of therapist inputs, interaction forces, and stride-to-stride adaptation during telerobotic locomotor training. *Left Panels:* Raw time-series data showing kinematics and forces across a single step for Patients 4 and 6 (P4 and P6). The positions of the manipulandum (dashed black line) and the robotic arm attachment point (solid black line) are shown. The measured interaction force ${\varvec{F}}_{{\varvec{R}}}$ (black line) reflects the actual force applied to the patient’s leg. The robotic controller tried to make ${\varvec{F}}_{{\varvec{R}}}$ match the trainer commanded force ${\varvec{F}}_{{\varvec{T}}}$ (red line), but the aggressiveness of the tracking was modulated according to the gait cycle. During the stance phase, a low-gain was used and was increased six-fold during the swing phase. The blue lines and arrows after heel-strike in the vertical position time-series highlight patient differences in the downward leg velocity at heel-strike; P4 hit the ground harder than P6, creating a sizeable vertical ${\varvec{F}}_{{\varvec{R}}}$. Note that trainer force transmission was software limited to be in the anterior and vertical directions; the trainer force was zeroed during a time-window starting at heel-strike and ending 200 ms later. *Right Panels:* Example of stride-to-stride adaptation in Patient 1 (P1). At the start of the trial, P1 walked without assistance (black diamonds), followed by several minutes of walking with assistance from a human trainer using the telerobotics system (red circles; the black trend lines are overlaid for illustrative purposes only)

The raw data from a single stride for two patients illustrate the operation of the telerobotics system in more detail (Fig. 7; left panel). The data show how for P4, the trainer deflected the manipulandum anteriorly during the early swing phase, which produced an anterior trainer force (${\varvec{F}}_{{\varvec{T}}}$) and the robotic arm applied a corresponding force ${\varvec{F}}_{{\varvec{R}}}$ to the patient’s leg. Note that the actual force applied by the trainer on the manipulandum (${\varvec{F}}_{{\varvec{M}}}$) was only a fraction of ${\varvec{F}}_{{\varvec{T}}}$ (e.g., if ${\varvec{F}}_{{\varvec{M}}} = 1.6$ N then ${\varvec{F}}_{{\varvec{T}}} = 30$ N). For the vertical force component, instead of targeting a specific phase of locomotion, the trainer lifted up on the manipulandum throughout the entire gait cycle, which translated into an upward force applied by the robotic arm. For P4, the force data show how the trainer force ${\varvec{F}}_{{\varvec{T}}}$ was zeroed after heel-strike to prevent a large oscillation in the robot-applied force (P4 exhibited a very large heel-strike impact). When force transmission restarted at mid-stance, there was a large tracking error until heel-off. This was by design, as the controller gains were lowered by a factor of six because the leg is typically stiff during stance, and high controller gains can induce oscillations. After heel-off, the controller gains were increased, and the measured force closely tracked the trainer force during most of the swing phase. For P6, the anterior–posterior manipulandum deflection did not exceed the deadband and therefore ${\varvec{F}}_{{\varvec{T}}}$ remained zero in this direction (Fig. 7, left panel). In the vertical direction, for P6 the trainer deflected the manipulandum upwards during only the swing phase, instead of the entire stride as done for P4. Also evident is the absence of a heel-strike oscillation in ${\varvec{F}}_{{\varvec{R}}}$ for P6, which is most likely because the downward velocity of the leg was lower (see blue slope annotation in the vertical position panel of Fig. 7, left panel), which, in turn, lessened the acceleration of the leg when the ground was contacted.

A more coarse-grained view of the raw data shows an example of patient adaptation over many steps (Fig. 7, right panel). The figure shows that after P1 walked for about 20 steps without assistance, the trainer began applying small assistive forces, which quickly increased in magnitude over a few steps. In response, P1 showed a small increase in swing height that persisted. In contrast, the changes in swing length were more gradual, more variable, and continued increasing throughout the trial. As the trial progressed, the trainer decreased the anterior assistive force while increasing the vertical force. For illustrative purposes, i.e., to highlight data trends, first-order discrete-time dynamic models (i.e., $x_{i + 1} = ax_{i}$, where $x$ is the state, $i$ is the step number, and $a$ is a coefficient) were fit to the patient swing kinematics (length and height) and fourth-order polynomials were fit to the trainer assistive forces (excluding initial steps with assistance).

#### Patient adaptation and retention

**Fig. 8:**
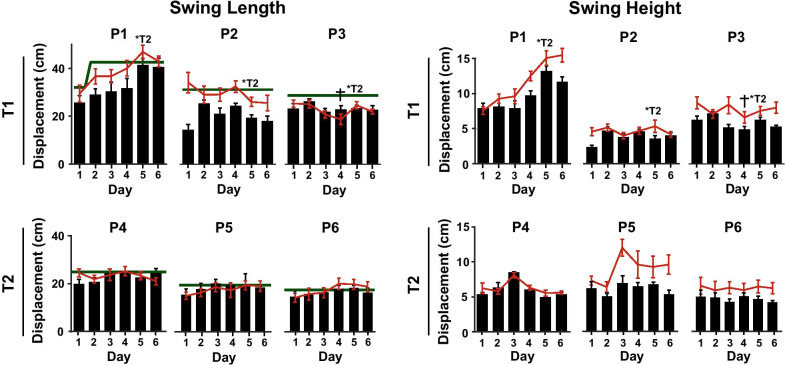
Adaptation and retention across 6 days of telerobotic locomotor training for six patients who have survived strokes (P1–P6). The black bars show the swing kinematics at the start of each day *without* assistance (average ± std. across steps for the first no-assistance trial). The red lines show the average swing kinematics at the end of the day *with assistance* from a trainer using the telerobotics system (average ± std. across steps for the last trial). P1–P3 received assistance from Trainer 1 (*except on Day 5 Trainer 2 assisted); P4–P6 received assistance from Trainer 2. ^†^P3 received resistance from Trainer 1 on Day 4. The step length target is indicated by the green horizontal line. For P1 the target was increased after the first day; for P2 the target was calculated using the third no-assistance trial. T1 = Trainer 1; T2 = Trainer 2

**Fig. 9:**
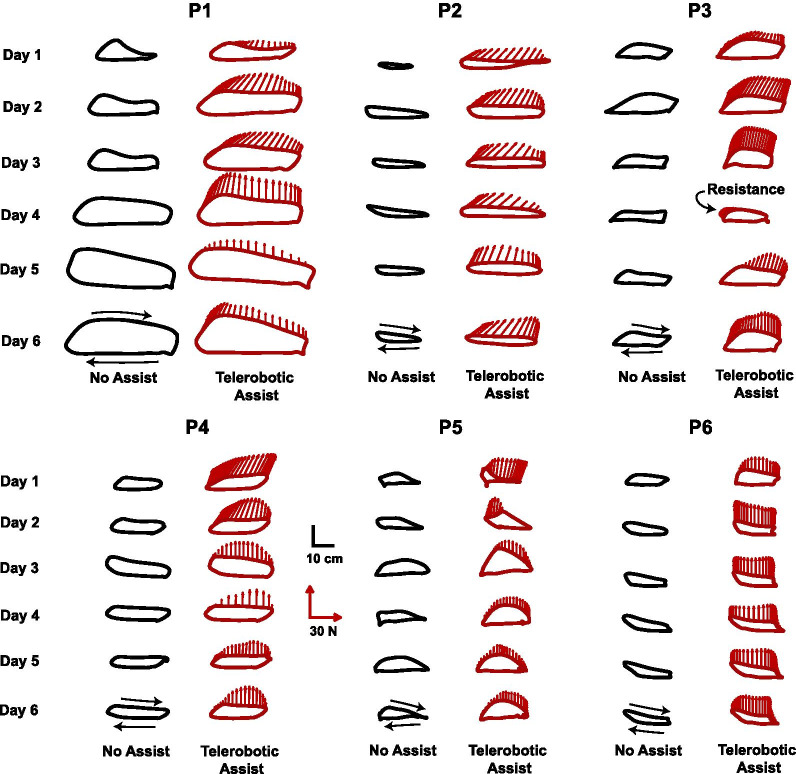
Average sagittal-plane leg trajectories and trainer forces for six patients who have survived strokes (P1–P6) across six practice days. For each patient, the black trajectories on the left show the average non-assisted leg trajectory during the initial trial of each practice day. The red trajectories in the right column show the average leg trajectory for the last trial of each day, in which the patient received assistance from a trainer using the telerobotic system. The red arrows show the average commanded trainer forces (${\varvec{F}}_{{\varvec{T}}}$) applied from heel-off to heel-strike. Trainer force transmission was software limited to be in the anterior and vertical directions, except P3 on Day 4, who received resistive forces

The average increase in unassisted swing length across practice (Day 1 vs. 6) was 4.1 ± 5.6 cm (mean ± standard deviation across subjects; p = 0.058). This equates to a 20 ± 21% increase, which was close to the 25% target increase. However, the variation between days did not follow a linear trend (Fig. 8). When comparing the maximum average daily change in unassisted swing length relative to Day 1, an increase of 7.2 ± 5.0 cm (p = 0.031) was observed. This equates to a 39 ± 23% increase in swing length. Unassisted swing height increased on average by 0.36 ± 1.8 cm (an 8 ± 31% increase; p = 0.752) between Days 1 and 6. The maximum average daily change in swing height was 2.0 ± 1.9 cm (a 41 ± 37% increase; p = 0.030). There was a substantial amount of variability between patients and from day to day in the unassisted and assisted kinematic trajectories (Fig. 9).

## Discussion

### Transparency of the telerobotics system

In robotic rehabilitation, the presence of significant uncontrolled forces creates challenges in understanding patient adaptive processes. The present study adds to prior research on the transparency of gait rehabilitation robots [39, 47, 54, 77]. The results showed that a magnetically-attached robotic arm that used end-effector force information to compensate for unwanted interaction forces could follow the legs of individuals walking on a treadmill, with forces averaging 3–4 N (swing–stance) for unimpaired participants and 2−3 N for patients who survived strokes (about 25–30% less). For five out of six patients, the interaction force during stance was tightly correlated with treadmill speed (R^2^ = 0.99), and the interaction force during swing had a moderate positive correlation with patient height (R^2^ = 0.71) and peak leg swing velocity (R^2^ = 0.55), Thus, for the patients, the existence of smaller unwanted interaction forces, compared to the unimpaired participants, may be related to their slower walking speeds. This suggests that the robot controller was more challenged at higher speeds, although the data cannot rule out stick–slip effects that might become significant at extremely slow speeds [73]. The weaker correlations during leg swing could be because there are fewer external constraints during the swing phase, during which individual differences in the neuromuscular control of gait may have a greater contribution to stride-to-stride locomotor variations.

The telerobotic system’s transparency was facilitated by a gain-scheduled proportional-integral force controller. The controller gains were locomotor-phase dependent, with high gains during the low-impedance swing phase of gait and low gains during the stance phase. Although the controller permitted adequate transparency overall, there were sometimes brief spikes in the interaction force near heel-strike. The magnitude of these forces varied. For individuals who survived strokes, some had no discernible impact peaks (e.g., P6 in Fig. 7), while others had significant impact peaks (e.g., P4 in Fig. 7). However, the effect of these brief disturbances was mitigated by the fact that they occurred while the leg was still on the treadmill and relatively stiff compared to the swing phase. More sophisticated algorithms could further improve transparency, such as adaptive model-based algorithms that predict unwanted interaction forces or disturbances [78, 79]. Performance could also be improved by optimizing the parameters of the robot controllers, such as through the use of artificial neural networks [80], fuzzy-logic [81], particle swarm optimization [82], or taking advantage of the cyclic nature of locomotion [83]. Finally, as pointed out by van Asseldonk and colleagues [47], there is no unambiguous standard for the level of transparency needed for robotic rehabilitation, which can only be determined by examining training outcomes of a more in-depth clinical trial.

How the presence of unwanted interaction forces may have altered the patients’ gait relative to walking without the robotic arm attached is at this time unknown, as kinematic measurements of patient locomotion without the robot arm attached were unavailable (the robot performed the measurements while attached). However, motion capture data were available for the two unimpaired individuals used in the preliminary transparency evaluation, which showed relatively minor effects (e.g., a 4.5 ± 2.3% decrease in swing length). Since the average interaction force magnitude was about 25–30% less in the patients, one would expect less of an impact on patient kinematics; however, it is difficult to extrapolate the transparency results to the full range of neuromotor impairments a patient might exhibit. In general, these transparency results are consistent with an earlier experiment by the investigators [49], which used the same robotic arm during treadmill locomotion, and reported minor gait deviations in unimpaired adults but did not report interaction forces. Interaction forces provide critical information related to robot transparency because a patient could compensate by increasing neuromuscular impedance while showing little change in locomotor kinematics.

### Patient and trainer outcomes

Six individuals who had survived strokes, who presented an asymmetric locomotor pattern with a shorter swing length on one side, successfully completed 6 days of locomotor training with the telerobotics system with the aim of increasing the affected leg’s swing length by 25%. All patients except P3 had larger unassisted swing lengths on the last day of training compared to the first day. The pooled data showed a 20% increase in swing length across training (p = 0.058). The learning trends were nonlinear; often, the last day was not a patient’s “best” day in terms of unassisted swing length. When comparing the unassisted swing length change between Day 1 and each patient’s best day, the increase was closer to 40% (p = 0.031). For most patients, the dominant assistive force provided by the trainers was upwards, which may assist with ground clearance and decrease the apparent weight of the leg during swing, which would, in turn, make it easier to swing the leg. Increased swing height was not a specific training goal, and the pooled patient data showed that unassisted swing height did not show consistent changes across practice. Based on the fact that all participants completed the training successfully with several demonstrating improvements in a targeted gait feature (swing length), it seems reasonable to conclude that the telerobotics approach is feasible. Future research should examine other outcome measures, such as gait symmetry and transfer to overground walking.

The first patient (P1) showed the greatest increase in swing length across training. However, P1 also had the highest walking speed, the largest baseline swing length, the longest time since the stroke event, and did not use an assistive device. Although, by these metrics, P1 was the least impaired, the amount of physical assistance provided to P1 during early training (especially, Days 2–4) was comparable to the other patients. However, the net assistance force provided to P1 decreased to only a few Newtons by the end of the training, while many of the other patients required substantial assistance throughout the training protocol. At this time, the degree to which the telerobotics approach’s effectiveness depends on the type and severity of locomotor impairment and the amount of assistance provided is unclear. This study was not designed to test efficacy, and the factors driving the observed locomotor changes cannot be teased apart. For instance, early gains could be due to increased familiarity with the treadmill and experimental setup. Improvements could also be driven by the exercise itself, such as increased cardiorespiratory fitness [10]. Adaptation processes in the trainers, as well as the switch in trainers for some patients (P1–P3), could have introduced additional variability into the patient learning outcomes.

The data showed that in several cases, the trainers tended to decrease assistance over the practice days. A trend for decreased assistance over time supports the foundational assumption of many “assist-as-needed” robotic gait training algorithms [54, 84, 85]. However, there were just as many instances where trainer assistance remained relatively constant or even increased slightly (e.g., P2 excluding Day 5 and P6). This could be related to slower rates of adaptation in more impaired patients and the limited 6-day training protocol. There were also marked differences between the two trainers. The second trainer (T2) provided much less force in the anterior–posterior direction, and in some cases, commanded practically zero force in this direction (e.g., P6). High between-trainer variability is supported by prior measurements of trainer-patient interaction forces during locomotor training [86]. More research is needed to identify the assistive strategies used by human trainers; a model-based system-identification approach could be useful in this regard [87]. Although the trainers were familiar with the operation of the telerobotics system (as study investigators), they were not professional therapists. Nevertheless, they were able to elicit targeted adaptations in many of the stroke survivors tested, which supports the feasibility of the system’s control architecture. Again, more research is needed to evaluate potential differences in how rehabilitation professionals interact with patients using the telerobotic system.

### Additional limitations, caveats, and future directions

This study differs conceptually from a pilot study, which typically resembles a miniature clinical trial and assesses broader patient outcomes and generalization of learning [88]. Because this study was mainly concerned with testing feasibility, a relatively high degree of variability in the physical capabilities and impairment characteristics of the patients was accepted. Broader conclusions about the efficacy of the telerobotics approach cannot be drawn without a larger sample, tighter inclusion/exclusion criteria, and adequate controls (e.g., manual “hands-on” assistance provided by a human therapist or assistance from an automated assist-as-needed algorithm). However, by design, the telerobotics system is ideally set up to facilitate such comparisons. Various assist-as-needed algorithms could be tested, and the approach eliminates an important confounder by allowing assistive forces to be delivered by either human trainers or an automated algorithm using the same physical interface. It would also be possible to test hybrid approaches. For example, data from the therapist and patient interactions could help create a customized algorithm that could take over for a human trainer. Although the transparency appeared sufficient, further work is needed to obtain a more robust evaluation, as the results may differ for other individuals, other speeds, and for different patient populations. Additional measures of transparency may also be informative, such as muscle activity [47, 89].

The present study’s telerobotics embodiment used a robotic arm magnetically attached to a rigid brace strapped onto the leg of a patient walking on a treadmill. Such end-effector robots cannot precisely control the configuration of an individual’s joints, which could be viewed as a limitation. Alternatively, as articulated by Sawers and Ting [90], although some patients may benefit from the added safety and confidence instilled by a robotic system that is highly-constrained and prescribes individual joint trajectories, the greater degree of joint-level variability afforded by an end-effector robot may promote sensorimotor recovery [68, 89]. While the single point of attachment limited the ability to measure the states of other body parts, such as the pelvis or contralateral leg, this could be overcome by adding a second robotic arm or using a motion capture system to record full-body kinematics. Finally, the robotic arm interface is treadmill-based, which is beneficial for training with body-weight support but may not fully transfer to overground walking. However, the telerobotics approach is not predicated on a particular piece of hardware, such as a robotic arm. For example, the manipulandum could control an exoskeleton, which could be worn by patients to facilitate overground walking.

More research is needed to explore the telerobotic approach’s feasibility with clinicians as operators and barriers associated with the adoption of the technology. For example, Liu and colleagues [91] showed that performance expectancy, which can be defined as “the degree to which an individual believes that using the system will help him or her to attain gains in job performance” [92], is the primary factor in determining technology adoption by rehabilitation professionals. In the present context, performance expectancy may relate to the extent that a clinician believes functional outcomes for patients would be improved. In addition to benefits associated with robotic augmentation, such as reduced trainer fatigue, the teleoperative capability of the system presents another benefit. A trained therapist could remotely feel the stiffness of an individual’s limbs, make judgments about musculoskeletal health, and apply forces to guide motor reeducation. This capability would be particularly beneficial for patients sensitive to the risks of disease transmission. However, due to communication delays, stability concerns can arise in long-distance teleoperation with bidirectional force application [93], which could be addressed with passivity-based control design [94–96], frequency-domain tools [97, 98], and human-in-the-loop adaptive control schemes [98, 99].

## Conclusions

The results support the feasibility of providing locomotor assistance to individuals who have experienced strokes using a telerobotics approach. However, a more comprehensive clinical study is needed with experimental controls before conclusions regarding efficacy can be made. The approach may also serve as a useful tool for testing hypotheses related to sensorimotor rehabilitation by virtue of its ability to measure the manipulative actions of human trainers, the motor responses of patients, and the forces associated with these interactions.

## Data Availability

The authors will make the data available upon request.
